# Supplementary material for: Impact of Particulate Matter on the Exacerbation of Immunoglobulin A Nephropathy: An Animal Experimental Study
Source: Int J Mol Sci. 2025 Sep 25;26(19):9387. doi: 10.3390/ijms26199387 (PMC12525145; doi:10.3390/ijms26199387)
Supplement: Supplementary file 1 [file ijms-26-09387-s001.zip › ijms-3794587-supplementary.pdf]

Supplementary Materials

Supplemental Table S1. GO and KEGG enrichment analysis of DEGs between HIGA-E and HIGA-NE.

(A.) GO enrichment results.

GO term	*Category*	*-log10(p-value)*
Nucleosome assembly	*Biological process*	*5.374*
Chromatin assembly	*Biological process*	*5.229*
Nucleosome organization	*Biological process*	*5.159*
Chromatin assembly or disassembly	*Biological process*	*5.132*
DNA packaging	*Biological process*	*4.871*
Protein–DNA complex assembly	*Biological process*	*4.708*
Chromatin remodeling	*Biological process*	*4.402*
Protein–DNA complex subunit organization	*Biological process*	*4.387*
DNA conformation change	*Biological process*	*4.031*
Negative regulation of myeloid cell differentiation	*Biological process*	*3.3*
DNA replication-dependent nucleosome assembly	*Biological process*	*3.258*
Regulation of myeloid cell differentiation	*Biological process*	*3.258*
Chromatin organization	*Biological process*	*2.703*
Immune system process	*Biological process*	*2.665*
Regulation of hemopoiesis	*Biological process*	*2.231*

Nucleosome	*Cellular component*	*10.171*
DNA packaging complex	*Cellular component*	*10.171*
Protein–DNA complex	*Cellular component*	*9.755*
Chromatin	*Cellular component*	*5.792*
Chromosome	*Cellular component*	*3.536*
Protein heterodimerization activity	*Molecular function*	*5.847*
Protein dimerization activity	*Molecular function*	*3.52*
DNA binding	*Molecular function*	*3.034*
RNA polymerase II core promoter sequence-specific DNA binding	*Molecular function*	*2.226*

(B.) KEGG pathway enrichment results.

Pathway	*Category*	*Gene count*	*Adjusted p-value*
Systemic lupus erythematosus	*KEGG*	*8*	*7.32e-11*
Neutrophil extracellular trap formation	*KEGG*	*8*	*5.46e-10*
Alcoholism	*KEGG*	*8*	*5.46e-10*
Viral carcinogenesis	*KEGG*	*4*	*7.13e-04*

Note: Downregulated pathways were predominant in HIGA-E compared with HIGA-NE.

Supplemental Table S2. Composition and particle size distribution of ISO 12103-1, A1 ultrafine test dust (Arizona test dust).

Component	Weight % (approx.)
Silicon dioxide (Quartz)	69–77%

Aluminum oxides	8–14%
Iron oxides	4–7%
Potassium oxides	2–5%
Calcium oxides	2.5–5.5%
Sodium oxides	1–4%
Magnesium oxides	1–2%
Titanium dioxide	0–1%

Supplemental Table S3. Particle size distribution of ISO 12103-1, A1 ultrafine test dust (Arizona test dust).

Particle size	Fraction (%)
< 0.97 μm	3–5%
< 1.38 μm	7–10%
< 2.75 μm	23–27%
< 5.50 μm	65–69%
< 11.0 μm	95.5–97.5%
< 22 μm	100%

Source: Product description of ISO 12103-1 Arizona test dust (DMT-Group, official specifications).

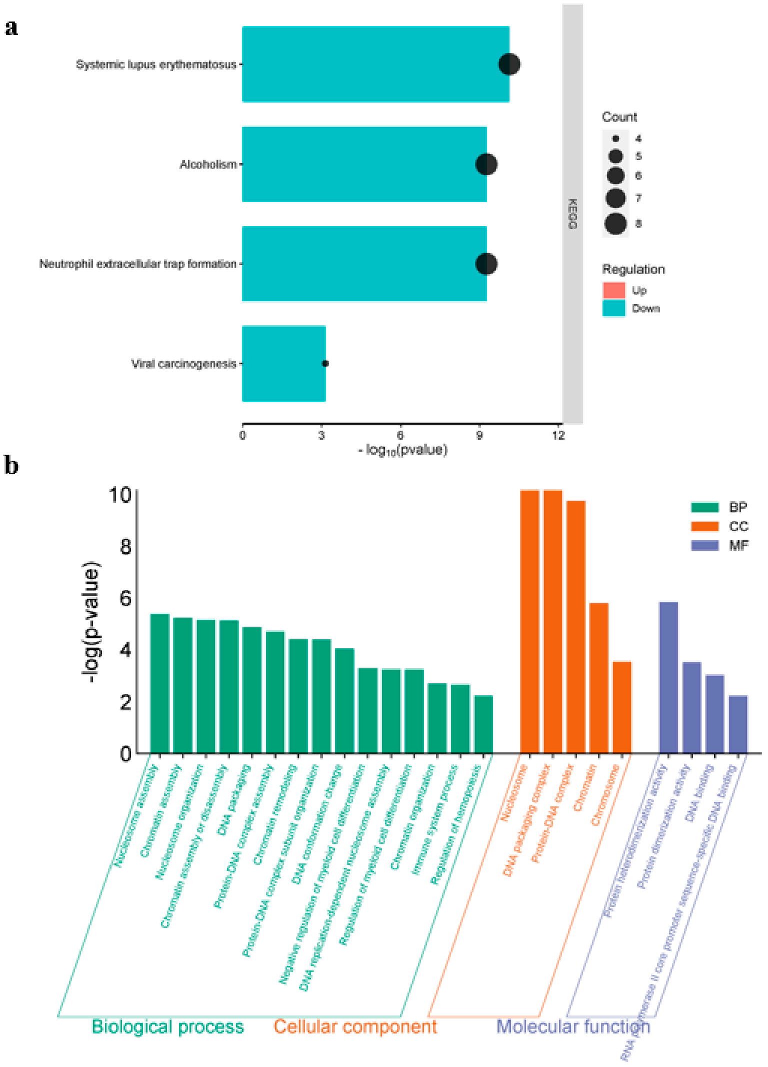

Supplemental Figure S1. Functional enrichment analysis of DEGs in splenic CD19⁺ B cells from HIGA mice following PM exposure. (**a.**) KEGG pathway enrichment analysis of downregulated genes in HIGA-E compared with HIGA-NE mice. Pathways such as systemic lupus erythematosus, neutrophil extracellular trap formation, and viral carcinogenesis were significantly enriched. (**b.**) GO enrichment analysis of DEGs in the same comparison. Downregulated genes were primarily associated with nucleosome assembly, chromatin organization, and DNA packaging, indicating altered genomic stability and epigenetic regulation.

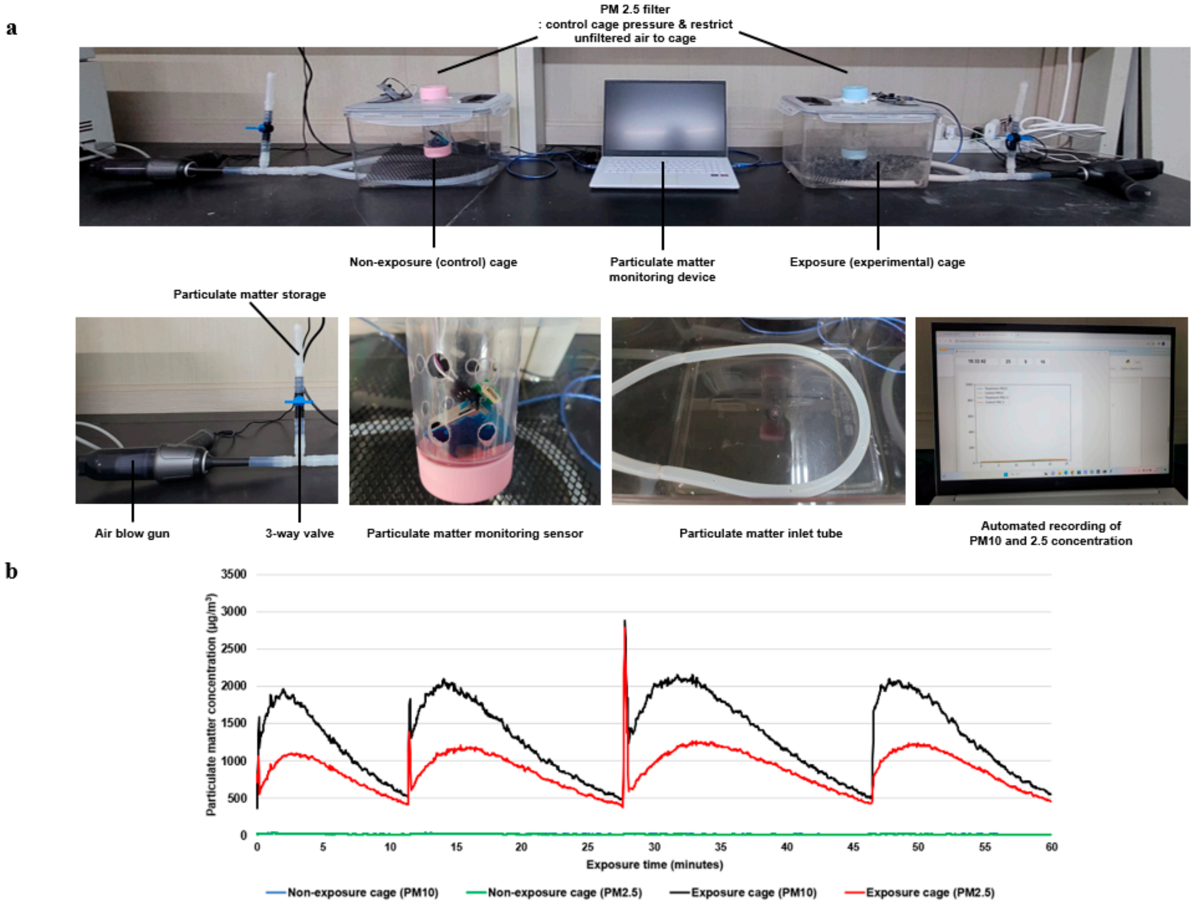

Supplemental Figure S2. PM exposure cage system and recording of particulate matter concentration. (**a.**) The sealed PM exposure cage and automated PM measurement systems are shown. The central laptop is connected to the PM measurement sensors, enabling real-time automated recordings of the PM concentrations. (**b.**) During PM exposure, the measurements indicate that the PM exposure cage maintains $PM_{2.5}$ and PM_{10} concentrations above a certain threshold. Additional PM is injected to sustain the target levels when the concentrations begin to decline. In contrast, the non-exposure cage maintains $PM_{2.5}$ and PM_{10} concentrations close to zero, as observed in the data.

The Python and Arduino scripts used in this study are available on GitHub (https://github.com/MinhyeokLee/PMmonitor).

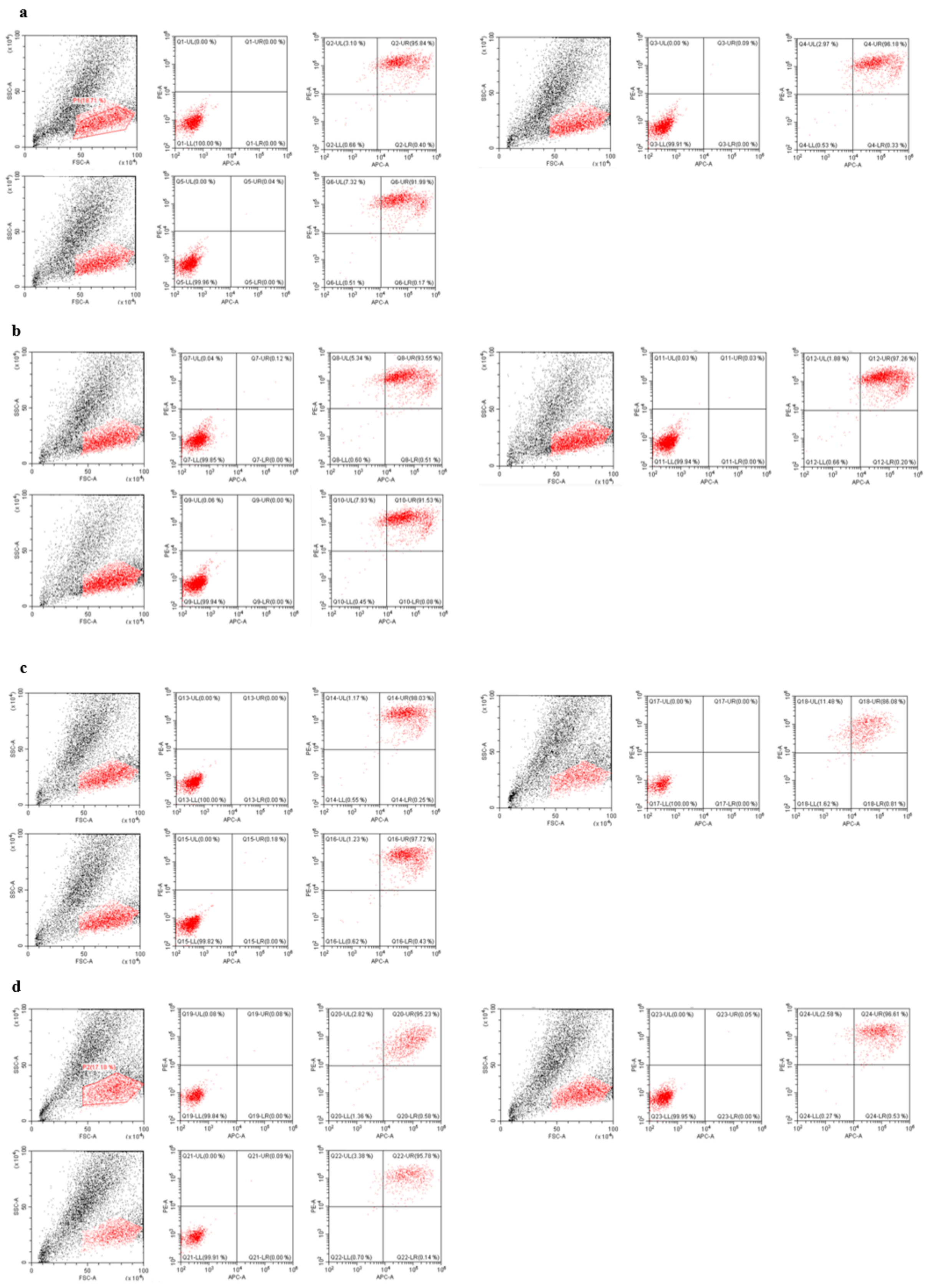

Supplemental Figure S3. Verification results of CD19+ splenic B cell separation using FACS. (**a.**) FACS validation results following CD19+ splenic B cell sorting from BALB/c-NE mice. (**b.**) FACS validation results following CD19+ splenic B cell sorting from BALB/c-E mice. (**c.**) FACS validation results following CD19+ splenic B cell sorting from HIGA-NE mice. (**d.**) FACS validation results following CD19+ splenic B

cell sorting from HIGA-E mice. The x-axis represents the CD19 marker, and the y-axis represents the CD20 marker. All samples demonstrated successful B cell separation with over 90% purity.
